# The Sun as a driver of the inner heliosphere: Modern investigations of fundamental plasma processes

**DOI:** 10.1038/s41467-026-72082-8

**Published:** 2026-05-11

**Authors:** Tamar Ervin, Aidan J. Nakhleh, Srijan Bharati Das

**Affiliations:** 1 Department of Physics, University of California, Berkeley, Berkeley, CA USA; 2 Space Sciences Laboratory, University of California, Berkeley, CA USA; 3 Department of Climate and Space Sciences and Engineering, University of Michigan, Ann Arbor, MI USA; 4 Center for Astrophysics, Harvard & Smithsonian, Cambridge, MA USA

**Keywords:** Solar physics, Astrophysical plasmas

## Abstract

The inner heliosphere serves as a natural laboratory to study fundamental plasma physics. A robust understanding of kinetic processes will fill gaps in our global understanding of the Sun and heliosphere, with applicability to laboratory and astrophysical plasma systems.

The Sun is our closest star, composed of superheated ionized gas called *plasma*. Currently a main-sequence star, it is nearly halfway through its life before transitioning to a red-giant phase. The Sun produces energy by fusing hydrogen atoms to helium in its core, a process that started about 4.6 billion years ago. This energy is propagated outwards as radiation until 0.7*R*_⊙_ and then through convective motion in a dynamic outer envelope. The solar atmosphere, lower corona, and our heliosphere are seeded by solar internal dynamics, which manifest across many orders of magnitude in spatial and temporal scales. Figure 1 shows an overview of the heliospheric system from the solar interior to near-Earth.

**Fig. 1:**
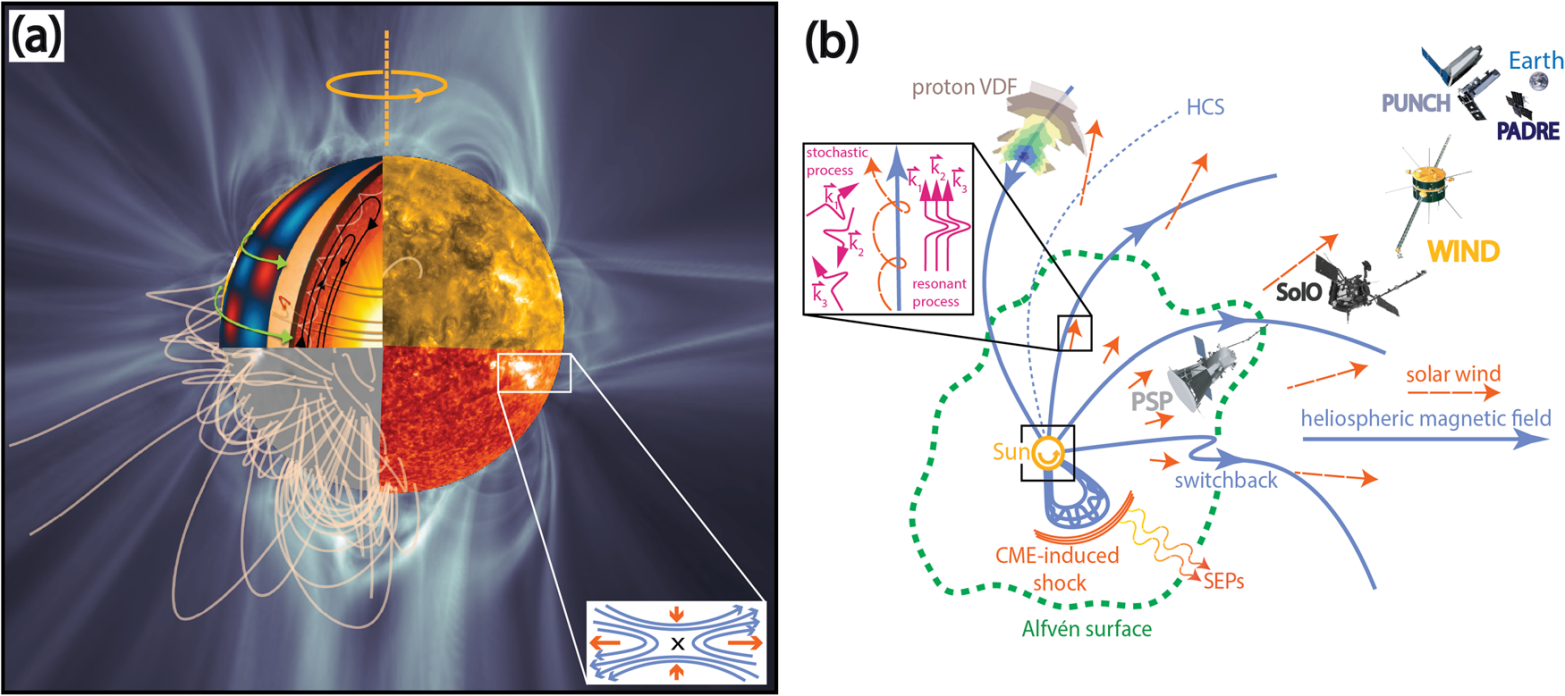
Overview of the Sun and inner heliospheric system (not to scale). Panel **a** shows a combination of models and observations in the near-Sun environment (black box region in the cartoon on the right surrounding the Sun). The *top left* quadrant is a model of the solar interior showing internal flow (differential rotation, meridional circulation probed via helioseismology), coupling with the magnetic field constituting the solar dynamo. The *bottom left* quadrant is a ground-based (GONG) observation of the photospheric magnetic field. Magnetic field lines from a Potential Field Source Surface (PFSS) extrapolation are overlaid. The right quadrant shows observations from the extreme ultraviolet imager (EUI) aboard Solar Orbiter in 174 Å (*top right*) and 304 Å (*bottom right*), which show emission from the corona and chromosphere, respectively. These are inset on a volume rendered Q-map (squashing factor), a quantification of the global magnetic field structure and separatrix web, from a time-dependent magnetohydrodynamic (MHD) simulation of the solar corona (Credit: Predictive Science Inc.^42^). Both the PFSS and MHD models used observations of the photospheric magnetic field as an inner boundary condition, an example of how observations drive coronal models. The bottom right of the inset shows a cartoon of magnetic reconnection associated with an active region structure. Panel **b** shows a heliospheric system overview of the inner-heliospheric spacecraft (discussed in this paper) taking remote and in-situ observations of the Sun and solar wind, alongside the Alfvén surface, a cartoon CME, switchback, solar wind outflow, HCS, and representative velocity distribution function (VDF, measured from PSP ^43^). We highlight some of the proposed heating mechanisms in the top left inset. We note that there are many more spacecraft from different space agencies making measurements that can help answer the open questions discussed in this comment; we chose to show the ones discussed in the text.

Solar plasma behaves like a perfect conductor, allowing fluid motions to advect, shear, and stretch magnetic fields, which drives the solar dynamo^1^. Decades of competing theoretical and computational frameworks have simulated the systematic 11-year solar cycle marked by the appearance of sunspots of opposite polarities within ~30^∘^ latitude, which migrate and vanish near the equator. The tachocline, a layer containing strong fossil magnetic fields and shear-flow operating at 0.7*R*_⊙_, is believed to be critical to the dynamo. A near-surface instabilities-driven dynamo^2^ has also been recently proposed. Dynamo simulations, used for predicting solar-cycle variations, are constrained by accurate knowledge of solar internal structure, flow, and magnetism. Helioseismologists have used decades of surface pulsation observations to estimate global features of structures, flows, and magnetism^3^.

Beyond the interior, the solar magnetic field expands outwards, driving the observed properties of the solar atmosphere. The visible solar surface, the photosphere, is characterized by granulation, sunspots, faculae, and emerging flux, with photospheric magnetic field observations used as inner boundary conditions for magnetic field extrapolations. Upwards from the photosphere, various topological structures in the chromosphere (prominences, filaments, plages, inward flows) and corona (coronal loops, coronal holes, streamers) are driven by the expanding magnetic field. There exists an unexplained multiple order of magnitude temperature jump from the photosphere and chromosphere (~6000–30,000 K) to the solar corona (~1–2 MK), known as the coronal heating problem. Magnetic reconnection has been proposed as one mechanism involved in this heating, efficiently transferring energy up through the solar atmosphere^4^, while recent evidence of small-scale Alfvén waves in the solar corona also points to their importance in the total energy budget^5^.

In the corona, thermal pressure gradient forces overcome solar gravity, leading to the expansion of the coronal magnetic field and plasma outflow known as the solar wind. Large-scale structures, such as the heliospheric current sheet (HCS) and streamers, are produced due to the configuration of the solar magnetic field, leading to magnetic reconnection and particle acceleration in the solar wind^6^. Observations have shown a non-adiabatic temperature fall off as the solar wind expands, indicating in-situ heating. On a large scale, these drivers have been identified: thermal pressure gradients of the particles, or driving from pressure gradients associated with Alfvénic fluctuations^7,8^. However, in both coronal and solar wind heating problems, a complete understanding of the collisionless dissipation processes is lacking.

As magnetic fields rise through the solar corona, turbulent motions beneath the photosphere bend and twist them, frequently building up enough free magnetic energy to trigger rapid field reconfiguration and energy release, which manifests as brief, intense local radiation bursts known as solar flares, and as eruptions of plasma into interplanetary space known as coronal mass ejections (CMEs)^9^. If these transients hit planetary magnetospheres, they can trigger strong space weather events, which may lead to detrimental impacts to technological infrastructure (e.g., GPS) in orbit and on the ground. Coronal models can be modified with initial conditions to trigger such transient events and simulate their evolution. Multi-spacecraft observations place constraints on simulation accuracy^10^, while evolving remote sensing capabilities strive to better resolve the initial conditions in the corona responsible for explosive solar transients^11^.

## Current objectives

Parker Solar Probe^PSP^^12,13^ is an inner heliospheric mission flying through the solar corona and measuring fields, particles, and coronal structure. Since its launch in 2018, PSP observations have provided insights into resonant heating of particles via ion- and electron-scale waves^14^ and the Alfvénic nature of solar wind turbulence^15^. Observational inference of the turbulent cascade rate confirmed the existence of a helicity barrier^16^, explaining preferential heating of ions. PSP has provided in-situ evidence that energetic particle acceleration near the Sun is driven by magnetic reconnection^6^ and shocks^17^. Further important breakthroughs include understanding type III radio bursts, which are signatures of the acceleration of non-thermal electron beams^18^, white-light imaging of large and small-scale coronal transients (e.g., streamers and CMEs^19^), and trajectory determination^20^ to better constrain coronal heating models.

At closest approach, PSP dips below 10 *R*_⊙_ allowing us to measure wind that is below the fragmented Alfvén surface^21^ where *v*_SW_ ~ *v*_A_. This “surface" separates the sub-Alfvénic region, where information can be transmitted in both directions from the Sun, from the super-Alfvénic region, where information can only be carried away from the Sun by the outward propagating solar wind. The geometry of stellar Alfvén surfaces has impacts on the angular momentum loss rate^22^ and potential habitability of exoplanetary worlds. Combining PSP in-situ and white light observations with those of the new PUNCH mission will allow for the study of the geometry and structure of this surface.

Recent work has shown differences in dissipation mechanisms between sub-Alfvénic and super-Alfvénic wind^23^. With prolonged spacecraft measurements below the Alfvén surface, we can probe coronal heating and its relation to solar wind heating, specifically studying how and whether the Alfvén surface modulates the efficacy of resonant versus non-resonant (stochastic) heating mechanisms. PSP observations have shown the prevalence of large amplitude Alfvénic fluctuations, or switchbacks, throughout the inner heliosphere, which have been shown to suppress reconnection and change turbulent dynamics^24^. There is debate over their origins: are they of solar origin^25^, or generated through in-situ processes^26^. Understanding their origin, evolution, and dissipation is critical and will rely on multi-point measurements through the inner heliosphere.

PSP aligns with other spacecraft in the Heliophysics fleet, resulting in observations of single solar wind streams multiple times in situ. These alignments allow for direct measurements of solar wind plasma evolution. Examples include relative increases in the kinetic energy of alpha particles to protons in the ambient solar wind^27^ and unprecedented views of transient event evolution, particularly solar energetic particle (SEP) propagation^28^. The diverse suite of multi-scale phenomena in solar plasmas^29^ drive current efforts to explore and constrain fundamental plasma processes such as species-dependent energization in the collisionless regime and the effect of magnetic field turbulence on energetic particle scattering and transport. These recent efforts illustrate the immense benefits of treating solar plasmas as natural laboratories to study kinetic plasma physics at resolutions far exceeding those obtained in terrestrial laboratories. In particular, investigations of wave-particle interactions and instabilities via PSP, Solar Orbiter, and Wind measurements of the evolution of electron and ion velocity distribution functions (VDFs) are crucial to probe energy conversion and dissipation in the collisionless regime^30^.

## Future outlook

Already at 25+ encounters around the Sun with a stable orbit perihelion of 9.8*R*_⊙_, PSP is perfectly poised to study in situ variability across solar cycles over the upcoming decades. In addition to understanding the impact of collisionless processes on electrons and protons, measurements of heavy ions are needed to understand the preferential heating that has been shown via simulations^31^. Strongly non-Maxwellian distributions^32^ observed in minor ion species are crucial in wave-particle interactions. Upcoming novel approaches to characterize these non-Maxwellian features are instrumental in bridging observation with theory^33^. With decades of ever-increasing high cadence observations from dozens of past, current, and upcoming missions, heliophysics has entered the big data era marked by a surge in machine learning applications for unsupervised detection and classification of transients, switchback, and energetic particle event characterization. Studies leveraging complementary multi-spacecraft observations and vantage points (e.g., ref. ^34^) have increasingly enabled source-to-downstream investigations of specific events across the heliosphere. Additionally, the solar poles and top-down views of the heliosphere remain an outstanding discovery opportunity in Heliophysics. The increase in Solar Orbiter’s inclination and a future 4*π* constellation (e.g., Firefly and Solaris) will allow us to image and study the polar regions to understand the generation and dynamics of the solar magnetic field and its impact on the solar wind and transient structures.

Identifying how energy is irreversibly dissipated in collisionless plasmas through the generation of entropy remains a longstanding challenge in plasma physics, partly due to the traditional view that entropy only increases from collisions. With the emergence of theories detailing collisionless entropy generation and non-Maxwellian relaxation in plasmas^35,36^, investigations of solar plasmas undergoing irreversible processes such as magnetic reconnection, collisionless shocks, and Alfvénic turbulence may help to inform and progress new frameworks of non-equilibrium thermodynamics. These observations may be paired with laboratory experiments of similar phenomena^37–39^. This connection is pertinent because in-situ solar wind instruments observe kinetic processes at high resolutions, but they are limited in their capacity to capture plasma evolution due to the general single-point nature of observations. In contrast, laboratory probes are inherently perturbative to the plasma and cannot peer into the kinetic regime as effectively as space plasma instruments, but the reproducibility of experiments allows for the plasma evolution to be constrained. By utilizing insights from laboratory experiments to interpret in situ solar wind measurements and vice versa, progress may be made to overcome the limitations of each environment, leading to new synergies between laboratory and space plasma physics. The proposed Collisionless High-beta Magnetized Experiment Researching Astrophysical Systems (CHIMERAS^40^) would serve as an excellent benchmark to explore this synergy further.

These dissipation and energy conversion processes require multi-point measurements to disentangle cross-scale coupling and understand energy transfer across boundaries, as single-point (with many assumptions) and four-point measurements are only able to provide information at one given spatial scale. Future missions, such as Helioswarm, will use nine spacecraft with separations from fluid to kinetic scales to investigate the 3D structure of magnetized turbulence^41^, while the proposed Plasma Observatory mission (seven spacecraft) will allow for the study of energy transport across plasma boundaries in the magnetosphere, connecting kinetic physics at work across different solar system bodies. In the future, new capabilities that standardize and miniaturize spacecraft components through small satellite and CubeSat missions utilizing commercial off-the-shelf technology (COTS) to provide space-qualified spacecraft buses (e.g., PADRE), provide pathways for development of affordable and scalable multi-spacecraft missions to make spatially separated measurements. In addition, funding for theoretical and modeling work is critical to understand modern space measurements and extrapolate those measurements both to future missions and to other less accessible plasma environments and astrophysical systems. The advancement of computational resources can support these efforts, as modeling the heliosphere requires resolving processes on a wide range of scales. Coupled models that describe and predict the impact of the solar wind on Earth’s magnetosphere are vital to planetary resilience against space weather. To fully close the loop, the impact of turbulence, collisionless dissipation, and kinetic physics must be included in these large-scale heliospheric models to account for these effects on the global system.

## Data Availability

Remote observations of the corona and chromosphere are from the EUI instrument on Solar Orbiter and are publicly available via soar.esac.esa.int/soar. The GONG magnetogram used for the potential field extrapolation can be accessed at gong.nso.edu and is an Open Access (OA) data product. Results of the time-dependent coronal magnetic field model used to produce the background map of the Sun in the Figure can be found at https://zenodo.org/records/14889337courtesy of Cooper Downs at Predictive Science Inc. The cartoon model of solar pulsations used in the Figure was adapted from Open Access visualizations by NASA/ESA.

## Code availability

The open access sunkit-magex package was used to produce potential field extrapolations.
